# Adverse effects of parental smoking during pregnancy in urban and rural areas

**DOI:** 10.1186/s12884-014-0414-y

**Published:** 2014-12-31

**Authors:** Helen Andriani, Hsien-Wen Kuo

**Affiliations:** International Health Program, National Yang-Ming University, Taipei, Taiwan; Institute of Environmental and Occupational Health Sciences, National Yang-Ming University, Taipei, Taiwan

## Abstract

**Background:**

Parental smoking during pregnancy is associated with lower birthweight and gestational age, as well as with the risks of low birthweight (LBW) and preterm birth. The present study aims to assess the association of parental smoking during pregnancy with birth outcomes in urban and rural areas.

**Methods:**

This was a secondary analysis of data collected in the Indonesia Family Life Survey, between 1993 and 2007, the first national prospective longitudinal cohort study in Indonesia. Retrospective data of parental smoking habits, socioeconomic status, pregnancy history and birth outcomes were collected from parents with children aged 0 to 5 years (n = 3789). We assessed the relationships between the amount of parental smoking during pregnancy with birthweight (LBW) and with gestational age (preterm birth).

**Results:**

We found a significant reduction in birthweight to be associated with maternal smoking. Smoking (except for paternal smoking) was associated with a decrease in the gestational age and an increased risk of preterm birth. Different associations were found in urban area, infants born to smoking fathers and both smoking parents (>20 cigarettes/day for both cases) had a significant reduction in birthweight and gestational age as well as an increased risk of LBW and preterm birth.

**Conclusions:**

Residence was found to be an effect modifier of the relation between parental smoking during pregnancy, amount of parental smoking, and birth outcomes on their children. Smoking cessation/reduction and smoking intervention program should be advised and prioritized to the area that is more prone to the adverse birth outcomes.

## Background

Tobacco smoking is a global public health problem. Cigarette smoke not only affects smokers but also contributes to the health problems of nonsmokers [1]. Birthweight in the short term increases when the frequency of parental smoking decreases; in the long term it increases when maternal health and nutrition improve [2]. Low birthweight (LBW) is associated with increased morbidities and mortalities in neonates. Preterm birth also leads to several complications, such as respiratory distress, feeding intolerance, and below-normal neurodevelopment [3].

Many chemicals in maternal smoking and second-hand smoke pass from the pregnant woman to the fetus through the placenta [4,5]. Nicotine, the most important component of tobacco, is present in the placenta at a 15% higher concentration than in maternal blood. Carbon monoxide in smoke can affect the baby’s growth and can lead to low birthweight [6]. Maternal active smoking during pregnancy induces birthweight decreases [7-9] and significantly increases the risk of LBW and preterm birth [10,11]. Additionally, when maternal and paternal cigarette consumption during pregnancy has been specifically evaluated relative to its association with birthweight and gestational age, the results are mixed. Many studies showed a significant decrease in the birthweight of their children among smoking fathers [11-16], while others demonstrated that paternal smoking did not decrease birthweight [17]. Some studies have suggested that the associations between paternal smoking with LBW and with preterm birth infants were insignificant [17-19].

The National Socio-Economic Survey and Basic Health Research, a series of population-based surveys in Indonesia, revealed that smoking prevalence among adults 15 years or older has increased from 26.9% in 1995, 31.5% in 2001, 34.4% in 2004, 34.2% in 2007 to 34.7% in 2010. This largely reflects an increasing smoking prevalence among males, from 53.4%, 62.2%, 63.1%, 65.6% to 65.9% during this period. As smoking among adult males has been steadily increasing, so too has consumption among women, from 1.7%, 1.3%, 4.5%, 5.2% to 4.2%, with almost a 300% increase from 1.3% in 2001 to 5.2% in 2007 [20-22]. Smoking prevalence rates in rural areas were higher than those in urban areas for both female and male adults [23]. If these smoking behaviors in female adults during pregnancy increase, the continued rise will inevitably lead to a further increase in the already high burden of birth outcomes on their children. This shows an urgent need to take action so as to reduce the prevalence of smoking.

In Indonesia, health disparities across the urban–rural gradient exist. Rural residents compared to urban, have poorer health outcomes and is one of influencing factors contributing to health inequalities in Indonesia [24]. Urban–rural disparities in adverse birth outcomes have been suggested including smoking behaviors [25,26]. In this study our primary objective was to evaluate the associations of existence of parental smoking and amount of parental smoking during pregnancy with the birth outcomes of their children of different residence.

## Methods

### Data sources

This study involved secondary analysis from Indonesia Family Life Survey (IFLS), the first national prospective longitudinal cohort study in Indonesia, collected between 1993 and 2007. The dataset are publicly available at RAND website (http://www.rand.org/labor/FLS/IFLS.html). The author granted access to the dataset by registering for access to the IFLS data download link. The sample represents about 83% of the Indonesian population, covering 13 major provinces out of a total of 27 provinces [27]. The IFLS randomly selected 321 enumeration areas (EAs) within each of the 13 provinces chosen from a nationally representative sample frame used in the 1993 SUSENAS, a socioeconomic survey. The SUSENAS frame, designed by the Indonesian Central Bureau of Statistics (BPS), was based on the 1990 census. Of the 7,730 households sampled, a complete interview was obtained for 7,039 households or 91.1 percent of households. In a household, two children aged 0 to 14 years were randomly selected. The eligible children included all biological, step or adopted children of the household head and spouse, as well as any children fostered to any adult in the household.

### Ethical oversight

The survey and its procedures were properly reviewed and approved by IRBs in the USA (at RAND) and in Indonesia, at Gajah Mada University (UGM) and earlier at University of Indonesia (UI). The relevant institutions should be able to accept the IRB approvals that were given for the surveys by RAND, UGM and UI and does not need to review any documents.

### Measurement

Parental smoking during pregnancy and birth outcomes were quantified between 1993 and 2007, using Indonesia Family Life Survey database. Parents with children aged 0 to 5 years gave information regarding their smoking habits, socioeconomic status, and pregnancy history and birth outcomes. There were 3,789 liveborn infants. Of these births, missing data included: birthweight 553 (14.6%) births and gestational age 553 (14.6%) births. These births were excluded from the study. In birth outcomes variables (birthweight and gestational age), 553 observations had missing data and were not included in the analysis. LBW means a birthweight of <2500 g, and preterm delivery was defined as a gestational age of <37 weeks.

Prenatal parental smoking was based on self-reported parental smoking history. The relevant questions were asked for mother and father separately, such as “Have you had the habit of smoking cigarettes? [Yes/no]”. “Do you still have the habit of smoking cigarettes or have you totally quit? [Still going on/have quit]”. We then constructed two variables: (a) paternal smoking status and (b) maternal smoking status. Each variable was classified into smokers, quitters, and non-smokers. To get information about the paternal and maternal smoking status during pregnancy, we pieced together information on “At what age (years) did you start to smoke cigarettes on a regular basis?” and “How long (years, months, weeks) have you totally quit from smoking cigarettes?” From the first question, we obtained information on whether the father or mother who smoked was smoking during pregnancy, by simultaneously looking at the age they began to smoke, their age, and their child’s age. From the second question, we obtained information on whether the father or mother who quit smoking smoked during pregnancy, by simultaneously looking at the duration of quitting smoking and their child’s age. We then constructed two variables: (a) paternal smoking during pregnancy and (b) maternal smoking during pregnancy. We classified each variable into smokers and non-smokers. We then combined paternal and maternal smoking during pregnancy into one key explanatory variable called “parental smoking during pregnancy”, which was categorized into: (a) neither parent smoked, (b) only the father smoked (paternal smoking), (c) only the mother smoked (maternal smoking); and (d) both parents smoked.

For parental smoking amount, the question was asked for mother and father separately, such as “How much do/did you approximately consume now/before totally quitting? [Total amount in cigarettes a day]”. We then constructed two variables: (a) paternal smoking amount and (b) maternal smoking amount. We classified paternal smoking into: (a) none, (b) 1-10 cigarettes/day, (c) 11-20 cigarettes/day, and (d) >20 cigarettes/day. Due to the small percentage of mothers who smoked > 10 cigarettes/day, maternal smoking amount was classified into three categories only: (a) none, (b) 1-10 cigarettes/day, and (c) >10 cigarettes/day. We then combined paternal and maternal smoking amount into one key variable called “smoking amount in both parents”. We considered three categories: (a) none, (b) 1-20 cigarettes/day, and (c) >20 cigarettes/day.

### Potential covariates and effect measure modifier

We considered the following as covariates for birthweight and gestational age: sex of infant (boy and girl), birth order (1, 2 or 3, and 4 or more), maternal age at delivery (<21, 21-34, and >34 years), father’s education (none, elementary, junior high school, senior high school, and post-graduate), maternal employment status (not working and working), parental BMI (both parents <25 kg/m^2^, only mother ≥ 25 kg/m^2^, only father ≥ 25 kg/m^2^, and both parents ≥25 kg/m^2^), household income (lowest, middle, highest), and residence (urban and rural). The information about the father’s education, maternal employment status, parental BMI, household income, and residence were obtained after the child was born. We used those variables as covariates for birthweight and gestational age because we assumed that those variables did not exhibit much difference before and after the child was born.

### Statistical analyses

We performed all statistical analyses using SPSS 20.0 and SAS 9.3 for Windows. We used Chi-squared tests to test for differences between parental smoking during pregnancy for each individual and family characteristic. Using multivariable linear regression, controlling for covariates, we assessed the relationships of parental smoking during pregnancy with continuous outcomes (birthweight and gestational age). Multiple logistic regression analyses controlled for covariates estimated the odds ratios (ORs) and 95% confidence intervals of LBW and preterm birth. To evaluate the functional relation between birth outcomes as a continuous and binary dependent variable and parental smoking amount, we applied a Generalized Additive Model (GAM). The models were adjusted for birth order. By default, the smoothing parameter for each B-spline term is chosen to yield four degrees of freedom. We set the statistical threshold for significance at 0.05.

### Sensitivity analyses

For sensitivity analyses, we re-ran the analyses to assess whether the birth outcomes results related to parental smoking would change substantially if we included/omitted adjustment to various covariates and interactions to ensure that they produce approximately the same results. We also tested the final model by including/omitting adjustment to various covariates to the stratified analysis. We found that a stratified model is sensitive to maternal age in the rural area but not in urban area.

## Results

Table 1 presents the distribution of basic characteristics of the participants and their families by parental smoking during pregnancy. Significant differences among parental smoking during pregnancy were identified for birth order, maternal age at delivery, father’s education, maternal employment, parental BMI, household income, and residence. We found the percentage of nonsmoking parents, paternal smoking, maternal smoking and both parents smoking during pregnancy to be 23.5%, 71.5%, 2.4%, and 2.7%, respectively. Compared to infants born to nonsmoking parents, infants born to smoking fathers were more likely to be born after the third child (26.8%); born to a mother younger than 21 years old (11.0%), a non-educated father (16.7%), or an unemployed mother (54.7%); born to parents both having a BMI < 25 kg/m^2^ (79.9%), a low income family (24.9%) or in a rural area (58.7%). We found similar results for infants born to smoking mothers and parents who both smoke (see Table 1).

**Table 1:** **Participants and their family characteristics and parental smoking during pregnancy**

**Characteristic**	**Neither parent smoked**	**Only father smoked**	**Only mother smoked**	**Both parents smoked**	***P***
	**n (%)**	**n (%)**	**n (%)**	**n (%)**	
Sex of infant					0.135
Boy	408 (51.3)	1220 (50.9)	43 (51.2)	89 (61.0)	
Girl	388 (48.7)	1176 (49.1)	41 (48.8)	57 (39.0)	
Birth order					0.001
1	201 (29.3)	544 (25.4)	17 (23.9)	15 (18.3)	
2 or 3	311 (45.3)	1022 (47.8)	32 (45.1)	28 (34.1)	
4 or more	175 (25.5)	574 (26.8)	22 (31.0)	39 (47.6)	
Maternal age at delivery					<0.001
<21years	67 (9.8)	235 (11.0)	17 (25.0)	7 (8.6)	
21 – 34 years	594 (87.2)	1834 (86.1)	46 (67.6)	66 (81.5)	
>34years	20 (2.9)	61 (2.9)	5 (7.4)	8 (9.9)	
Father’s education					<0.001
None	126 (16.6)	380 (16.7)	19 (23.5)	33 (22.9)	
Elementary	285 (37.6)	1169 (51.3)	27 (33.3)	73 (50.7)	
Junior high	105 (13.9)	300 (13.2)	8 (9.9)	22 (15.3)	
Senior high	152 (20.1)	309 (13.6)	19 (23.5)	12 (8.3)	
Post-graduate	89 (11.8)	121 (5.3)	8 (9.9)	4 (2.8)	
Maternal employment					0.015
Not working	388 (53.0)	1224 (54.7)	37 (48.7)	45 (39.8)	
Working	344 (47.0)	1015 (45.3)	39 (51.3)	68 (60.2)	
Parental BMI					<0.001
Both parents < 25 kg/m^2^	510 (74.5)	1703 (79.9)	52 (73.2)	68 (84.0)	
Only mother ≥ 25 kg/m^2^	83 (12.1)	268 (12.6)	7 (9.9)	12 (14.8)	
Only father ≥ 25 kg/m^2^	66 (9.6)	126 (5.9)	8 (11.3)	0 (0.0)	
Both parents ≥ 25 kg/m^2^	26 (3.8)	35 (1.6)	4 (5.6)	1 (1.2)	
Household income					0.001
Q1 (lowest)	119 (17.8)	504 (24.9)	12 (18.2)	23 (26.4)	
Q2 (middle)	391 (58.6)	1154 (57.1)	43 (65.2)	43 (49.4)	
Q3 (highest)	157 (23.5)	364 (18.0)	11 (16.7)	21 (24.1)	
Residence					<0.001
Urban	401 (50.4)	990 (41.3)	41 (48.8)	43 (29.5)	
Rural	395 (49.6)	1406 (58.7)	43 (51.2)	103 (70.5)	

Birth order, maternal age at delivery, maternal employment status, parental BMI, and residence were significantly associated with parental smoking during pregnancy. Table 2 shows that after adjusting for the covariates, we found the mean birthweight to be significantly associated with paternal and maternal smoking. Newborns of parents with a nonsmoking father and smoking mother had a mean birthweight of 136 lower than that of newborns of nonsmoking parents. Infants born to smoking parents decreased 84 g, on an average, though this result was not statistically significant (p = 0.096). With the baseline of nonsmoking parents, the odds ratios for paternal smoking (OR = 0.89), maternal smoking (OR = 2.58), and both parents smoking (OR = 2.37) did not show an association with a risk of LBW.

**Table 2:** **Parental smoking during pregnancy correlated with birthweight (g) and low birthweight**

	**Linear regression**		**Logistic regression**	
	**Birthweight**	***p***	**Low birthweight**	***p***
	**Mean ± SE**		**OR (95% CI)**	
Parental smoking during pregnancy^a^				
Neither parent	3057 ± 16.2	-	1.00	-
Only father	3094 ± 9.1	0.047	0.89 (0.51-1.54)	0.665
Only mother	2921 ± 52.2	0.013	2.58 (0.75-8.90)	0.135
Both parents	2973 ± 47.6	0.096	2.37 (0.51-10.92)	0.270
Paternal smoking amount^b^				
None	3051 ± 16.2	-	1.00	-
1-10 cigarettes/day	3108 ± 14.2	0.009	0.81 (0.58-1.14)	0.230
11-20 cigarettes/day	3094 ± 14.1	0.044	0.66 (0.46-0.94)	0.022
>20 cigarettes/day	2900 ± 28.8	<0.001	2.09 (1.38-3.17)	<0.001
Maternal smoking amount^b^				
None	3085 ± 8.7	-	1.00	-
1-10 cigarettes/day	2968 ± 51.2	0.025	1.66 (0.84-3.27)	0.144
>10 cigarettes/day	2734 ± 76.4	<0.001	4.07 (1.88-8.82)	<0.001
Smoking amount in both parents^b^				
None	3067 ± 16.9	-	1.00	-
1-20 cigarettes/day	3095 ± 10.0	0.154	0.95 (0.69-1.31)	0.747
>20 cigarettes/day	2911 ± 26.4	<0.001	2.24 (1.47-3.41)	<0.001

Note. SE = standard error; OR = odds ratio; CI = confidence interval.

^a^Adjusted for birth order, maternal age at delivery, maternal employment, parental BMI, and residence.

^b^Adjusted for birth order.

Due to small percentage of mothers who smoked and both parents who smoked, we only put birth order as a covariate for the association between parental smoking amount and birth outcomes of their children. The association between paternal smoking amount and birthweight was evident in the group of fathers who smoked >20 cigarettes/day, with a 151 g decrease in birthweight. The association of the maternal smoking amount with birthweight and risk of LBW appeared to be dose-dependent. All infants of the maternal smoking groups had lower birthweights, ranging from 117 g (1-10 cigarettes/day) to 351 g (>10 cigarettes/day). The largest estimate of association between the smoking amount in both parents and birthweight was among parents who smoked > 20 cigarettes/day (combined), with an average birthweight decrease of 156 g. The fathers who smoked >20 cigarettes/day had a significantly higher risk of having LBW infants than those fathers who did not smoke (OR = 2.09, 95% CI [1.38, 3.17], *p* < 0.001). Mothers who smoked > 10 cigarettes per day had a significant fourfold increase in LBW risk compared with nonsmoking mothers (OR = 4.07, 95% CI [1.88, 8.82], *p* < 0.001). Compared with the nonsmoking parents, the risk of LBW significantly increased in for parents who (combined) smoked >20 cigarettes/day (OR = 2.24, 95% CI [1.47, 3.41], *p* < 0.001).

Table 3 shows that after adjusting for the covariates, we found infants with smoking mothers and those with both parents smoking to have significantly shorter at gestational ages than those with nonsmoking parents. Newborns of parents with nonsmoking fathers and smoking mothers were born at a significant deficit, 0.96 weeks sooner than that of newborns of nonsmoking parents. Infants born to both smoking parents were born 0.58 weeks sooner, on average, than those born to nonsmoking parents (*p* = 0.006). Compared with the nonsmoking parents, children with smoking mothers were 3.37 times more likely to be born at preterm gestation (OR = 3.37, 95% CI [1.58, 7.19], *p* = 0.002). Children with smoking parents were 3.10 times more likely to be born at preterm gestation than those with nonsmoking parents (OR = 3.10, 95% CI [1.37, 7.04], *p* = 0.007).

**Table 3:** **Parental smoking during pregnancy correlated with gestational age (weeks) and preterm birth**

	**Gestational age**	***p***	**Preterm birth**	***p***
	**Mean ± SE**		**OR (95% CI)**	
Parental smoking during pregnancy^a^				
Neither parent	39.79 ± 0.1	-	1.00	-
Only father	39.75 ± 0.1	0.595	1.16 (0.78-1.71)	0.470
Only mother	38.83 ± 0.2	<0.001	3.37 (1.58-7.19)	0.002
Both parents	39.21 ± 0.2	0.006	3.10 (1.37-7.04)	0.007
Paternal smoking amount^b^				
None	39.64 ± 0.1	-	1.00	-
1-10 cigarettes/day	39.93 ± 0.1	<0.001	0.51 (0.34-0.75)	0.001
11-20 cigarettes/day	39.76 ± 0.1	0.144	0.78 (0.55-1.11)	0.163
>20 cigarettes/day	39.20 ± 0.1	0.001	2.11 (1.38-3.23)	0.001
Maternal smoking amount^b^				
None	39.78 ± 0.1	-	1.00	-
1-10 cigarettes/day	39.06 ± 0.2	<0.001	2.68 (1.45-4.95)	0.002
>10 cigarettes/day	38.77 ± 0.3	<0.001	5.54 (2.61-11.76)	<0.001
Smoking amount in both parents^b^				
None	39.73 ± 0.1	-	1.00	-
1-20 cigarettes/day	39.82 ± 0.1	0.231	0.86 (0.61-1.20)	0.373
>20 cigarettes/day	39.26 ± 0.1	<0.001	2.30 (1.49-3.56)	<0.001

Note. SE = standard error; OR = odds ratio; CI = confidence interval.

^a^Adjusted for birth order, maternal age at delivery, father’s education, household income, and residence.

^b^Adjusted for birth order.

The association between the paternal smoking amount and gestational age was evident in the group of fathers who smoked >20 cigarettes/day, with a significantly decrease in gestational age (0.44 weeks). The association of maternal smoking amount with gestational age and the risk of preterm birth appeared to be dose-dependent. The maternal smoking group had shorter gestational ages, ranging from 0.72 weeks (1-10 cigarettes/day) to 1.01 weeks (>10 cigarettes/day). The largest estimated association between the smoking amount in both parents and gestational age was found in the group of >20 cigarettes/day, with a significant decrease in gestational age (0.47 weeks). The association between paternal smoking and preterm birth was more obvious in infants whose fathers smoked >20 cigarettes/day (OR = 2.11, 95% CI [1.38, 3.23], *p* = 0.001). The risks of preterm birth were significantly higher in mothers who smoked 1-10 cigarettes per day (OR = 2.68, 95% CI [1.45, 4.95], *p* = 0.002) and >10 cigarettes per day (OR = 5.54, 95% CI [2.61, 11.76], *p* < 0.001). With the nonsmoking group as the baseline, the risk of preterm birth increased with the number of cigarettes smoked daily for both parents > 20 cigarettes/day (OR = 2.30, 95% CI [1.49, 3.56], *p* < 0.001).

From the stratified analyses in Table 4, after adjusting for the covariates, mean birth weight was significantly associated with both parents smoking in urban area and maternal smoking in rural area. The mean birth weight of infants born to smoking fathers was 60 g higher (*p* = 0.053) in urban area and 16 g higher (*p* = 0.168) in rural area than that of infants born to nonsmoking parents. The mean birth weight of infants born to smoking mothers was 14 g lower (*p* = 0.878) in urban area and 262 g lower (*p* < 0.001) in rural area than that of infants born to nonsmoking parents. The mean birth weight of infants born to smoking parents was 186 g lower (*p* = 0.049) in urban area and 28 g lower (*p* = 0.608) in rural area than that of infants born to nonsmoking parents. However, no significant associations were found between LBW and parental smoking during pregnancy in four groups.

**Table 4:** **Parental smoking during pregnancy correlated with birth weight (g) and low birth weight, stratified by residence**

	**Urban**				**Rural**			
	**Linear regression**		**Logistic regression**		**Linear regression**		**Logistic regression**	
	**Birthweight**	***p***	**Low birthweight**	***p***	**Birthweight**	***p***	**Low birthweight**	***p***
	**Mean ± SE**		**OR (95% CI)**		**Mean ± SE**		**OR (95% CI)**	
Parental smoking during pregnancy^a^								
Neither parent	3062 ± 26.3	-	1.00	-	3057 ± 19.8	-	1.00	-
Only father	3122 ± 16.4	0.053	0.58 (0.27-1.26)	0.168	3073 ± 10.3	0.489	1.48 (0.67-3.28)	0.332
Only mother	3048 ± 86.3	0.878	0.78 (0.08-8.12)	0.834	2795 ± 63.2	<0.001	5.91 (0.98-35.67)	0.053
Both parents	2876 ± 90.6	0.049	0.93 (0.11-7.90)	0.947	3029 ± 51.9	0.608	8.77 (0.94-81.47)	0.056
Paternal smoking amount^b^								
None	3081 ± 25.2	-	1.00	-	3018 ± 20.9	-	1.00	-
1-10 cigarettes/day	3151 ± 23.4	0.044	0.78 (0.46-1.33)	0.361	3074 ± 17.4	0.041	0.79 (0.50-1.23)	0.296
11-20 cigarettes/day	3103 ± 26.1	0.551	0.94 (0.55-1.60)	0.809	3090 ± 16.0	0.006	0.49 (0.30-0.78)	0.003
>20 cigarettes/day	2850 ± 47.4	<0.001	2.89 (1.59-5.27)	0.001	2940 ± 35.4	0.058	1.52 (0.85-2.71)	0.160
Maternal smoking amount^b^								
None	3105 ± 14.7	-	1.00	-	3069 ± 10.3	-	1.00	-
1-10 cigarettes/day	2913 ± 86.8	0.029	1.41 (0.49-4.10)	0.524	3012 ± 61.2	0.359	1.94 (0.80-4.69)	0.143
>10 cigarettes/day	2806 ± 154.9	0.055	1.13 (0.14-9.01)	0.905	2704 ± 82.1	<0.001	6.23 (2.60-14.92)	<0.001
Smoking amount in both parents^b^								
None	3078 ± 26.4	-	1.00	-	3053 ± 21.9	-	1.00	-
1-20 cigarettes/day	3127 ± 17.3	0.117	0.87 (0.55-1.40)	0.577	3073 ± 11.9	0.431	0.98 (0.62-1.53)	0.911
>20 cigarettes/day	2855 ± 44.5	<0.001	2.66 (1.46-4.84)	0.001	2954 ± 31.9	0.010	1.90 (1.04-3.46)	0.036

Note. SE = standard error; OR = odds ratio; CI = confidence interval.

^a^Adjusted for birth order, maternal age at delivery, maternal employment status, and parental BMI.

^b^Adjusted for birth order.

The mean birth weight of infants born to fathers who smoked >20 cigarettes/day was 231 g lower (*p* < 0.001) in urban area and 78 g lower (*p* = 0.058) in rural area than that of infants born to nonsmoking fathers. The association of maternal smoking amount with birth weight and risk of LBW appeared to be dose-dependent in both urban and rural areas, ranging from -192 g (1-10 cigarettes/day) to -299 g (>10 cigarettes/day) in urban area and from -57 g (1-10 cigarettes/day) to -365 g (>10 cigarettes/day) in rural area. The mean birth weight of infants born to the smoking amount in both parents >20 cigarettes/day were 223 g lower (*p* < 0.001) in urban area and 99 glower (*p* = 0.010) in rural area than that of infants born to nonsmoking parents. The odds ratios (ORs) for LBW of infants born to fathers who smoked >20 cigarettes/day were 2.89 (*p* = 0.001) in urban area and 1.52 (*p* = 0.160) in rural area, respectively. The OR for LBW of infants born to mothers who smoked >10 cigarettes/day in urban and rural area was 1.13 (*p* = 0.905) and 6.23 (*p* = <0.001), respectively. The ORs for LBW of infants born to the smoking amount in both parents >20 cigarettes/day were 2.66 (*p* = 0.001) in urban area and 1.90 (*p* = 0.036) in rural area, respectively.

From the stratified analyses in Table 5, after adjusting for the covariates, infants with both parents who smoked in urban area and mothers who smoked in rural area were significantly shorter at gestational age than those with nonsmoking parents. Compared to infants born to nonsmoking parents, the mean gestational age of infants born to smoking mothers was 0.62 weeks shorter (*p* = 0.086) in urban area and 1.31 weeks shorter (*p* = <0.001) in rural area. The mean gestational age of infants born to smoking parents was 1.09 weeks shorter (*p* = 0.003) in urban area and 0.2 weeks shorter (*p* = 0.440) in rural area. The ORs for preterm birth of infants born to smoking mothers were 1.87 (*p* = 0.307) in urban area and 5.14 (*p* = 0.003) in rural area, respectively. The ORs for preterm birth of infants born to smoking parents were 4.70 (*p* = 0.002) in urban area and 1.49 (*p* = 0.638) in rural area, respectively.

**Table 5:** **Parental smoking during pregnancy correlated with gestational age (weeks) and preterm birth, stratified by residence**

	**Urban**				**Rural**			
	**Gestational age**	***p***	**Preterm birth**	***p***	**Gestational age**	***p***	**Preterm Birth**	***p***
	**Mean ± SE**		**OR (95% CI)**		**Mean ± SE**		**OR [95% CI]**	
Parental smoking during pregnancy^a^								
Neither parent	39.69 ± 0.1	-	1.00	0-	39.84 ± 0.1	-	1.00	-
Only father	39.54 ± 0.1	0.219	1.16 (0.69-1.95)	0.582	39.91 ± 0.1	0.482	1.13 (0.61-2.08)	0.704
Only mother	39.07 ± 0.4	0.086	1.87 (0.56-6.18)	0.307	38.53 ± 0.3	<0.001	5.14 (1.72-15.41)	0.003
Both parents	38.60 ± 0.4	0.003	4.70 (1.74-12.73)	0.002	39.64 ± 0.2	0.440	1.49 (0.28-7.82)	0.638
Paternal smoking amount^b^								
None	39.61 ± 0.1	-	1.00	-	39.66 ± 0.1	-	1.00	-
1-10 cigarettes/day	39.73 ± 0.1	0.339	0.76 (0.46-1.26)	0.286	40.10 ± 0.1	<0.001	0.29 (0.15-0.55)	<0.001
11-20 cigarettes/day	39.56 ± 0.1	0.722	0.96 (0.57-1.60)	0.869	39.88 ± 0.1	0.038	0.68 (0.42-1.11)	0.120
>20 cigarettes/day	38.90 ± 0.2	0.001	2.80 (1.56-5.01)	0.001	39.45 ± 0.1	0.195	1.60 (0.85-2.98)	0.143
Maternal smoking amount^b^								
None	39.60 ± 0.1	-	1.00	-	39.91 ± 0.0	-	1.00	-
1-10 cigarettes/day	38.62 ± 0.3	0.002	3.11 (1.37-7.06)	0.007	39.41 ± 0.2	0.038	2.16 (0.83-5.64)	0.115
>10 cigarettes/day	38.85 ± 0.6	0.184	4.34 (1.12-16.87)	0.034	38.74 ± 0.3	<0.001	6.76 (2.73-16.77)	<0.001
Smoking amount in both parents^b^								
None	39.64 ± 0.1	-	1.00	-	39.81 ± 0.1	-	1.00	-
1-20 cigarettes/day	39.64 ± 0.1	0.974	0.89 (0.56-1.40)	0.615	39.94 ± 0.1	0.167	0.88 (0.52-1.46)	0.613
>20 cigarettes/day	38.92 ± 0.2	<0.001	2.79 (1.56-4.98)	0.001	39.53 ± 0.1	0.068	1.97 (1.02-3.82)	0.045

Note. SE = standard error; OR = odds ratio; CI = confidence interval.

^a^Adjusted for birth order, maternal age at delivery, father’s education, and household income.

^b^Adjusted for birth order.

After adjusting for birth order, the mean gestational age of infants born to fathers who smoked >20 cigarettes/day was 0.71 weeks shorter (*p* = 0.001) in urban area and 0.21 weeks shorter (*p* = 0.195) in rural area than that of infants born to nonsmoking fathers. The association of maternal smoking amount with gestational age appeared to be dose-dependent in rural area, but not in urban area. All infants of the maternal smoking groups had shorter gestational age, ranging from 0.75 weeks (>10 cigarettes/day) to 0.98 weeks (1-10 cigarettes/day) in urban area and from 0.5 weeks (1-10 cigarettes/day) to 1.21 weeks (>10 cigarettes/day) in rural area. The largest estimate effect of the smoking amount in both parents on gestational age was found among group of >20 cigarettes/day, decreased in gestational age of 0.72 weeks (*p* < 0.001) in urban area and -0.28 weeks (*p* = 0.068) in rural area. The ORs for preterm birth of infants born to fathers who smoked >20 cigarettes/day were 2.80 (*p* = 0.001) in urban area and 1.60 (*p* = 0.143) in rural area, respectively. There was a dose-dependent association between maternal smoking amount and risk of preterm birth both in urban and rural areas. The ORs for preterm birth of infants born to mothers who smoked 1-10 cigarettes/day were 3.11 (*p* = 0.007) in urban area and 2.16 (*p* = 0.115) in rural area, respectively and >10 cigarettes/day in urban and rural area were 4.34 (*p* = 0.034) and 6.76 (*p* < 0.001), respectively. The ORs for preterm birth of infants born to both parents’ smoking amount >20 cigarettes/day were 2.79 (*p* = 0.001) in urban area and 1.97 (*p* = 0.045) in rural area, respectively.

Table 6 showed the nonparametric component of the model. The chi-square values indicate that for birthweight and LBW, the smoothing terms of paternal smoking amount and smoking amount in both parents are highly significant, and maternal smoking amount is not statistically significant. For gestational age and preterm birth, the chi-square values indicate that the nonparametric effect of paternal smoking amount, maternal smoking amount, and smoking amount in both parents are significant at the 5% level.

**Table 6:** **Results for estimation of Generalized Additive Model (GAM) to birth outcomes, adjusted for birth order**

**Variables**	**Parameter estimates**		**Analysis of deviance**	
	**Estimate**	**Standard error**	**Chi-square**	***p***
Birthweight				
Paternal smoking amount	−2.097	0.900	30.09	<0.001
Maternal smoking amount	−16.454	3.732	6.14	0.105
Smoking amount in both parents	−2.890	0.890	27.67	<0.001
Gestational age				
Paternal smoking amount	−0.010	0.004	22.67	<0.001
Maternal smoking amount	−0.062	0.014	11.14	0.011
Smoking amount in both parents	−0.013	0.004	16.87	0.001
Low birthweight				
Paternal smoking amount	0.017	0.006	17.75	<0.001
Maternal smoking amount	0.070	0.019	1.18	0.554
Smoking amount in both parents	0.022	0.006	12.23	0.002
Preterm birth				
Paternal smoking amount	0.021	0.007	16.07	<0.001
Maternal smoking amount	0.088	0.019	6.55	0.038
Smoking amount in both parents	0.028	0.007	10.45	0.005

**Figure 1:**
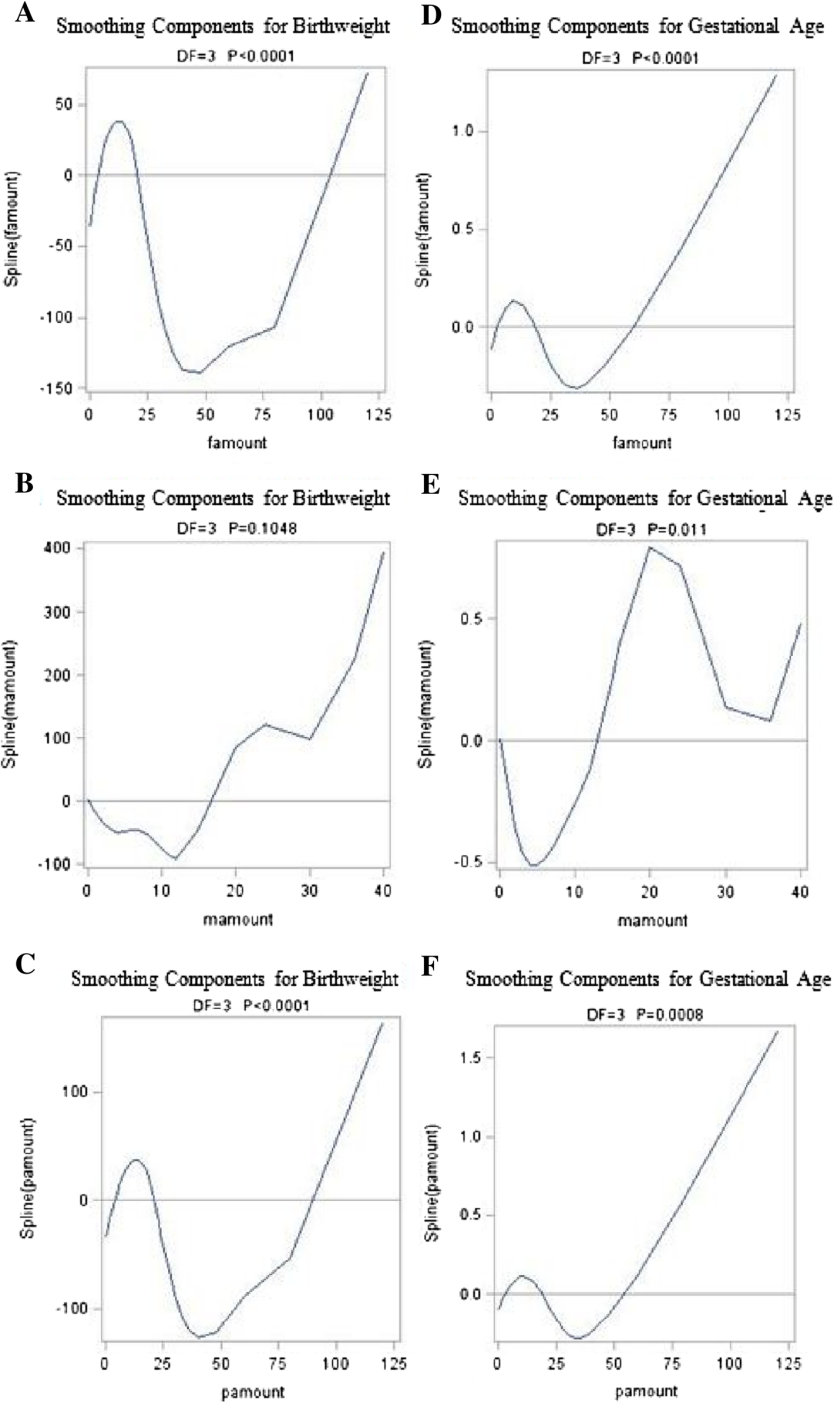
**GAM-estimated relationships between parental smoking amount and birthweight (A-C) and gestational age (D-F), adjusted for birth order.** Note. famount = paternal smoking amount; mamount = maternal smoking amount; pamount = smoking amount in both parents.

Figure 1 graphically shows the relationships between parental smoking amount and birth outcomes as the continuous variables. The birthweight increased sharply approximately below 15 cigarettes/day (Figure 1A and 1C), decreased sharply above this number, and then shows a rapid increasing response when the smoking amount rise above 50 cigarettes/day. Figure 1B shows a sharp decreased response below approximately 10 cigarettes/day, and then a rapid increasing response above this number; however the association was not statistically significant. Figure 1D and 1F shows a modest increased response below 12 cigarettes/day, a modest decreased above this number, and then a gradual increasing response when the smoking amount rise above approximately 35 cigarettes/day. The gestational age decreased gradually with maternal smoking amount below 5 cigarettes/day (Figure 1E), increased sharply above this number, decreased sharply above 20 cigarettes/day, and then shows an increasing response when the smoking amount rise above 35 cigarettes/day.

**Figure 2:**
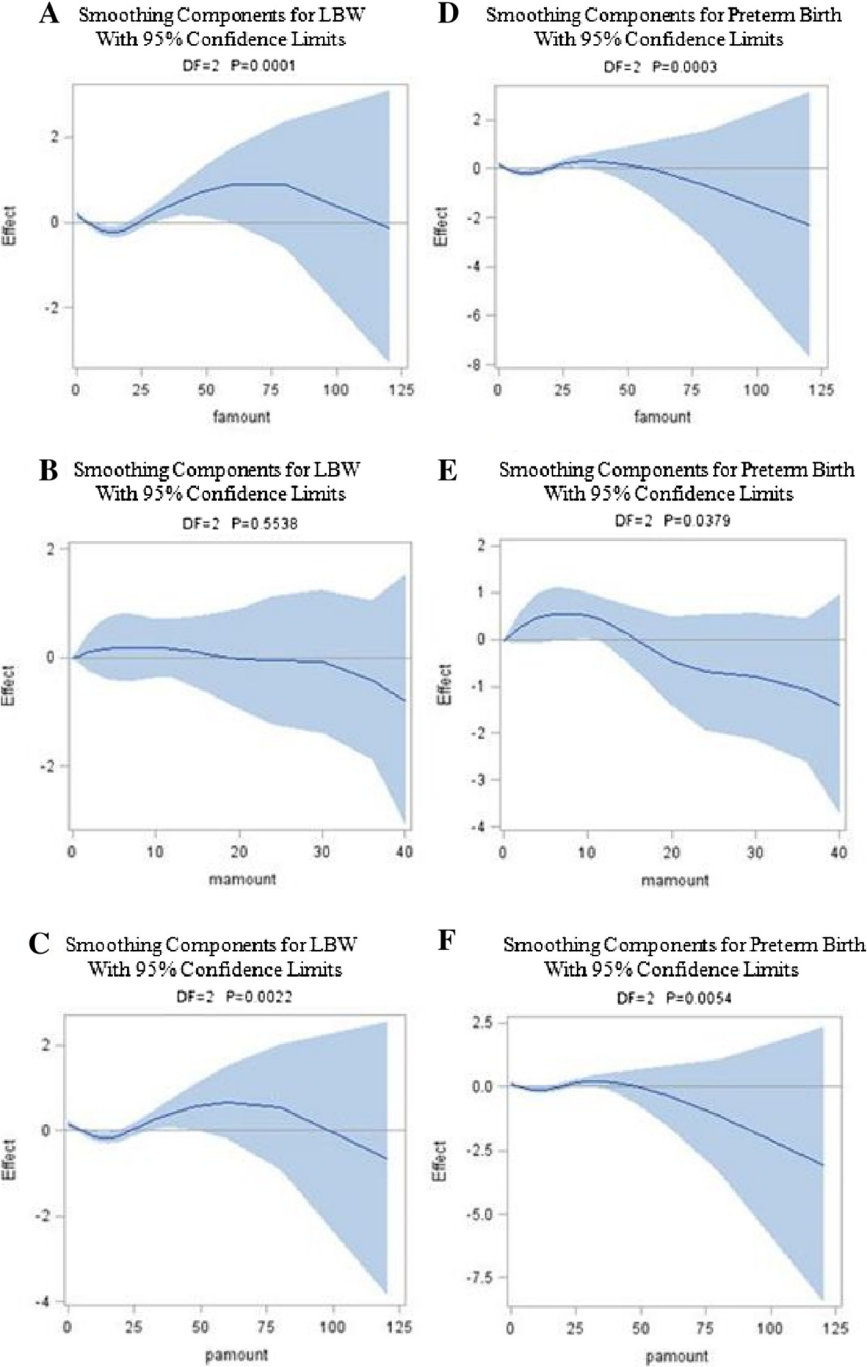
**GAM-estimated relationships between parental smoking amount and LBW (A-C) and preterm birth (D-F), adjusted for birth order.** The solid lines indicate the estimated mean percentage of change in birth outcomes, and the dotted lines represent 95% confidence interval. Note. famount = paternal smoking amount; mamount = maternal smoking amount; pamount = smoking amount in both parents.

Figure 2 depicts the relationships between parental smoking amount and birth outcomes, defined as binary indicators (LBW and preterm birth). Figure 2A and 2C shows almost zero response of LBW to paternal smoking amount and smoking amount in both parents below 25 cigarettes/day, a modest increased response above this number, showing a peak at approximately 75 cigarettes/day after which the response decline. Figure 2B depicts a negative relationship between LBW and maternal smoking amount; however the association was not statistically significant. Figure 2D and 2F shows almost zero response of preterm birth to paternal smoking amount and smoking amount in both parents below 50 cigarettes/day after which the response declines. Preterm birth increases gradually with rising maternal smoking amount (Figure 2E), showing a peak at approximately 10 cigarettes/day after which the response declines. All plots become highly variable at the ends.

## Discussion

### Associations of parental smoking during pregnancy with the birth outcomes of their children

We have presented representative population-based data concerning child birthweight and gestational age in Indonesia. Our study confirms the detrimental associations between parental smoking during pregnancy and birth outcomes. Previous studies have shown a significant birthweight decrease among infants born to smoking fathers that decrease ranging from 11-113 g [11-16]. Second-hand smoke contains many toxic chemicals that can harm an unborn baby. They are inhaling the nicotine, carcinogens, and toxic chemicals found in tobacco smoke [4]. Nicotine and carbon monoxide in the blood of a pregnant woman exposed to second-hand smoke can decrease the blood flow to the unborn baby, affect the heart, lungs, digestive system, and central nervous system and may lead to low birthweight [28]. However, in our study we found that paternal smoking did not decrease birthweight. This result is in line with a previous study [17]. Our explanation is that fathers might choose to not smoke in the presence of their pregnant wives. Although previous studies have supported our findings and described an insignificant association of paternal smoking with LBW and preterm birth infants [17-19], current studies indicates that paternal and maternal smoking create significant adverse effects in LBW and the preterm birth of newborns.

Although the prevalence of maternal smoking was relatively low in our study, the maternal smoking groups revealed a significantly lower mean birthweight, shorter mean gestational age, and a higher risk of preterm birth than did the nonsmoking parents groups. But maternal smoking was not associated with the risk of LBW. Our finding is consistent with previous studies demonstrating that active maternal smoking during pregnancy induces birthweight and gestational age reduction [7-9,29,30]. The associations of maternal smoking during pregnancy with LBW and with preterm birth were greater and statistically significant [10,11,31-33]. Animal studies [28] also demonstrate how maternal smoking affects birthweight through the effects of nicotine on fetal and placental development [34]. The flow of blood between the uterus and the placenta is slowed by nicotine [35], which restricts the supply of nutrients and oxygen. Smoke produces carbon monoxide, which is potent vasoconstrictors of placental vessels, combines with oxygen to carry hemoglobin to form carboxyhemoglobin, thus further restricting the supply of oxygen. A consequence of these mechanisms is lower fetal growth, which in turn can lower birthweight and increases preterm births.

The results of this study show that both parents smoking groups showed a non-significant lower mean birthweight and a higher risk of LBW than did the nonsmoking parents groups due to limited sample size. Previous reports showed that birthweight of infants was found to be further reduced if both parents smoked [12]. Yerushalmy [36] found that there was a significant increase in the LBW rate when both parents smoked, but not when only one parent smoked, compared with when neither smoked. The insignificant association between both parents smoking and the risk of LBW was due to low percentage of both parents smoking (2.7%) and low proportion of LBW (8.3%). In our study, compared with nonsmoking parents, both parents smoking were significantly correlated with shorter mean gestational age and a higher risk of preterm delivery. We suggested that maternal smoking is more influential than paternal smoking in relation to gestational age and preterm birth. Previous studies on maternal smoking have supported this finding [29-33] and demonstrating that maternal smoking was significantly associated with shorter gestational age and a risk of preterm birth.

### Associations of parental smoking amount during pregnancy with birth outcomes on their children

In our study, paternal smoking amount played an important role on the infant birthweight, especially fathers who smoked >20 cigarettes per day. Our value of 151 g compares well with previous studies demonstrating that newborns of nonsmoking mothers whose fathers smoked >20 cigarettes per day had a mean deficit of 88 g [37] and an average reduction in birthweight of 120 g [38]. Previous reports from Italy [39] and India [40] also suggest significant associations of paternal smoking on birthweight. Our findings, those fathers who smoked >20 cigarettes/day had a higher risk of getting LBW infants and shorter at gestational age compared with those born to non-smoking fathers, were also in line with the study in Japan [13]. Amounts of maternal smoking revealed a lower mean birthweight and gestational age as well as a much higher OR of LBW and preterm birth than did the nonsmoking mothers and this association showed a dose-dependent trend. In previous studies, a difference in risk between heavy and light smokers was consistently reported [41]. Compared with those born to non-smoking mothers, the birthweight notably decreased with advancing number of cigarettes smoked per day: being doubled OR with statistically significance of LBW. These findings are in essentially parallel with the result of meta-analysis by Kramer [42]. Moore and Zaccaro [43] also found different risks for different numbers of cigarettes smoked per day, but, in a systematic review and meta-analysis, this relationship was not clear [44]. Both mother-smokers and father-smokers showed the same effect as contemporary international data [2]. The effect of father’s smoking would appear greater when the women admitted to smoking (presumably truthfully), possibly because if the father smokes, the mother is likely to smoke more heavily [38]. These facts could perhaps be used to persuade new parents to reduce their smoking.

We furthermore used an analytical approach (Generalized Additive Modelling) that is specifically designed to analyse data when the impact of predictors on the outcome variables are nonlinear. We showed that paternal smoking amount, maternal smoking smoking, and smoking amount in both parents were all closely associated with birth outcomes, except for maternal smoking amount, which was not associated with birthweight and LBW. The sharp decrease in birthweight between 15-50 cigarettes/day and in gestational age between 12-35 cigarettes/day as well as the gradual increased in LBW between 25-75 cigarettes/day and in preterm birth between 1-50 cigarettes/day for smoking fathers and both smoking parents provides a partial explanation that heavy cigarette smoking can endanger unborn babies. The shortened gestational age between 1-5 cigarettes/day and between 20-35 cigarettes/day as well as the modest increased in preterm birth between 1-10 cigarettes/day for smoking mothers also suggest that even a light cigarette smoking can have an outsize effect on the pregnancy duration.

### Associations of parental smoking and amount of parental smoking during pregnancy with birth outcomes in urban and rural areas

Our study confirms different detrimental effects on birth outcomes of smoking during pregnancy by different residence. Paternal smoking did not decrease birth weight and did not have any significant influences on the LBW, gestational age, or preterm birth of their children in both urban and rural area. Previous studies have supported our finding [17-19]. The explanation is that fathers may try not to smoke in the presence of pregnant wives thus the newborns or infants do not directly expose from fathers’ smoking. It is possible that the average amount of cigarettes smoking per day in fathers were not high enough to observe an effect on adverse birth outcomes in their children. In urban area, the associations between fathers who smoked >20 cigarettes/day and the smoking amount in both parents >20 cigarettes/day with birth weight, gestational age, and the risk of LBW and preterm birth were significant; however in rural area, the associations were not significant. The national nutritional surveillance system among more than 175,000 urban slum households reported that paternal smoking predicts an increased probability of short-term and chronic child malnutrition [45]. The environment condition and social and family networks in urban area such as more densely populated, weaker relationships and interactions of the people, and higher rate of air pollution from vehicles, industry, domestic heating, and cooking sources which exceed the capacity of the cities’ natural ventilation systems, more smoke-free environments from public area, may also contribute to adverse birth outcomes in urban area compare to rural area.

Maternal smoking, especially mothers who smoked >10 cigarettes/day was not significantly associated with birth weight, gestational age, and the risk of LBW and preterm birth in urban area, but the associations were significant in rural area. There was a significant higher percentage of smoking mothers, non-educated mothers, working mothers, low family income in rural area than that in urban area. This is consistent with the nationally representative surveys in Indonesia showed that female adults smoking prevalence was higher in rural areas than the urban areas [22,23,46]. A research study specifically found that women living in rural areas were at significantly increased risk for having preterm and LBW infants [47]. The higher level of smoking prevalence in rural areas was probably due to the low level of knowledge of the health risks of smoking, less information about smoking hazards, in addition to more poverty and lower levels of educational attainment compared to those living in urban areas [48]. Women of low socioeconomic status are at increased risk for delivering LBW babies [49]. Research has shown that poverty, both at the regional and household level, predicts LBW and preterm birth [50,51]. Previous studies have revealed that women without a high school diploma are at significantly higher risk of delivering preterm, LBW, and small-for-gestational-age babies [51,52]. Low-income families have a tendency to consume a less nutritious diet than other households because they do not consume the recommended amounts of fruit, vegetables, meats, whole grains and low-fat dairy products. Mothers of a low income family feel the need to improve their economy by working. People in rural area are mostly having homogenous profession and depending on agriculture. Despite the beneficial effects of employment on income, mothers who work in strenuous occupations, including those that involve prolonged standing, are at heightened risk for both preterm delivery and having LBW babies [53]. Occupational exposures to toxic substances and solvents have also been linked to preterm delivery [54-56]. Further, rural women are more likely to be underserved by prenatal and obstetric care that can prevent pregnancy complications [57]. Unintended pregnancy can lead to poor birth outcomes through its association with risky behaviors such as prenatal smoking and unhealthy weight maintenance, higher prevalence of which have been demonstrated for rural women [51,58].

### Strengths and limitations

Numerous studies have demonstrated that maternal smoking and/or maternal exposure to passive smoke during pregnancy is associated with adverse birth outcomes, however further evidences for smoking behaviors by both parents on birth outcomes have rarely been analyzed. There is insufficient scientific research on smoking and health in the Indonesian population. To our knowledge, this study is among the first studies in Indonesia to assess the association of paternal smoking, maternal smoking, and both parents smoking during pregnancy with birth outcomes on their children in urban-rural settings.

The findings of the present study should be viewed with some limitations in mind. First, reports of parental smoking during pregnancy were retrospective. While studies show high reliability in self-reported smoking status [59], nonetheless, the extent to which under-reporting of smoking status may or may not occur is unknown, thereby introducing some non-differential misclassification bias. Second, birthweight and gestational age were obtained from maternal recall or self-reported rather than hospital birth records. Although maternal recall of birthweight and gestational age seems to be sufficiently accurate for clinical and epidemiological use [60,61], the accuracy of self-reported birthweights is less certain. Some mothers were unsure of the date of their last menstruation (perhaps due to period irregularities), therefore they reported their pregnancy duration in months without detailed information about its weeks. Previous studies have reported a poor degree of correspondence between birthweights recorded in official records and self-reported birthweights [62]. Third, the dissemination of information on adverse consequences of smoking may have discouraged some mothers or fathers from disclosing it. It is likely that many mothers or fathers who smoked claimed to have stopped smoking. Lastly, the information about father’s education, maternal employment status, parental BMI, household income, and residence were obtained after the child was born. Socioeconomic and lifestyle change as well as urban-rural migration, however, may have influenced our findings to some extent.

## Conclusions

A significant reduction in birthweight and gestational age, as well as an increased risk of LBW and preterm birth were found to be associated with maternal smoking and the heavy amount of parental smoking. Smoking cessation/reduction should be advised to pregnant women to reduce morbidities in their neonates. In urban areas, there continues to be a clear need for health care professionals to counsel both smoking expectant mothers with smoking spouses, especially when their husbands smoked >20 cigarettes/day regarding the adverse consequences of smoking during their pregnancy and offer behavioral counseling. Not only does maternal smoking during pregnancy, directly and indirectly, influence the well-being of the children (from fetal stages through young adulthood) but also has key health consequences for the expecting mother.

## References

[1] Das SK (2003). Harmful health effects of cigarette smoking. Mol Cell Biochem.

[2] Alberman E (1991). Are our babies becoming bigger?. J R Soc Med.

[3] Behrman RE, Butler AS (2007). Preterm Birth: Causes, Consequences, and Prevention Institute of Medicine Committee on Understanding Premature, Birth Assuring Healthy, Outcomes The National Academies Collection: Reports funded by National Institutes of Health.

[4] World Health Organization: **Tobacco smoke and involuntary smoking.** *IARC Monogr Eval Carcinog Risks Hum* 2004, **83**:1-1438. PMC4781536 15285078

[5] Office on S, Health (2006). Publications and Reports of the Surgeon General. The Health Consequences of Involuntary Exposure to Tobacco Smoke: A Report of the Surgeon General.

[6] Office on S, Health (2006). Publications and Reports of the Surgeon General. The Health Consequences of Smoking-Nicotine Addiction: A Report of the Surgeon General.

[7] Abel EL (1980). Smoking during pregnancy: a review of effects on growth and development of offspring. Hum Biol.

[8] D’Onofrio BM, Turkheimer EN, Eaves LJ, Corey LA, Berg K, Solaas MH, Emery RE (2003). The role of the children of twins design in elucidating causal relations between parent characteristics and child outcomes. J Child Psychol Psychiatry.

[9] Schell LM, Hodges DC (1985). Variation in size at birth and cigarette smoking during pregnancy. Am J Phys Anthropol.

[10] Bada HS, Das A, Bauer CR, Shankaran S, Lester BM, Gard CC, Wright LL, Lagasse L, Higgins R (2005). Low birth weight and preterm births: etiologic fraction attributable to prenatal drug exposure. J Perinatol.

[11] Ward C, Lewis S, Coleman T (2007). Prevalence of maternal smoking and environmental tobacco smoke exposure during pregnancy and impact on birth weight: retrospective study using millennium cohort. BMC Public Health.

[12] Campbell MJ, Lewry J, Wailoo M (1988). Further evidence for the effect of passive smoking on neonates. Postgrad Med J.

[13] Matsubara F, Kida M, Tamakoshi A, Wakai K, Kawamura T, Ohno Y (2000). Maternal active and passive smoking and fetal growth: a prospective study in Nagoya, Japan. J Epidemiol.

[14] Misra DP, Nguyen RH (1999). Environmental tobacco smoke and low birth weight: a hazard in the workplace?. Environ Health Perspect.

[15] Windham GC, Hopkins B, Fenster L, Swan SH (2000). Prenatal active or passive tobacco smoke exposure and the risk of preterm delivery or low birth weight. Epidemiology.

[16] Zhang J, Ratcliffe JM (1993). Paternal smoking and birthweight in Shanghai. Am J Public Health.

[17] Underwood PB, Kesler KF, O’Lane JM, Callagan DA (1967). Parental smoking empirically related to pregnancy outcome. Obstet Gynecol.

[18] Ko TJ, Tsai LY, Chu LC, Yeh SJ, Leung C, Chen CY, Chou HC, Tsao PN, Chen PC, Hsieh WS (2014). Parental smoking during pregnancy and its association with low birth weight, small for gestational age, and preterm birth offspring: a birth cohort study. Pediatr Neonatol.

[19] Lin YJ (2014). Low birth weight, preterm births, and intrauterine growth retardation in relation to parental smoking during pregnancy. Pediatr Neonatol.

[20] Barber S, Ahsan A, Adioetomo SM, Setyonaluri D (2008). Tobacco Economics in Indonesia.

[21] Dorotheo U: **Cigarette Tax and Price : Affordability and Impacts on Consumption and Revenue.** In: *Cigarette Affordability and Impact of Tobacco Taxes in Indonesia.* Jakarta: Demographic Institute, Faculty of Economics, University of Indonesia; 2011.

[22] Republic of Indonesia MoH (2004). The Tobacco Source Book: Data to Support a National Tobacco Control Strategy.

[23] Suhardi (1997). Perilaku Merokok di Indonesia. Seri Survei Kesehatan Rumah Tangga, Departemen Kesehatan, Republik Indonesia.

[24] Wood J: **Old problems, fresh solutions: Indonesia’s new health regime.** In: *Economist Intelligence Unit.* Edited by Line D; 2010.

[25] Bailey BA, Cole LK (2009). Rurality and birth outcomes: findings from southern appalachia and the potential role of pregnancy smoking. J Rural Health.

[26] Strutz KL, Dozier AM, van Wijngaarden E, Glantz JC (2012). Birth outcomes across three rural-urban typologies in the finger lakes region of New York. J Rural Health.

[27] Christine EP (2000). Documentation for IFLS1-RR: Revised and Restructured 1993 Indonesian Family Life Survey Data, Wave 1. RAND.

[28] Centers for Disease C, Prevention, National Center for Chronic Disease P, Health P, Office on S, Health (2010). Publications and Reports of the Surgeon General. How Tobacco Smoke Causes Disease: The Biology and Behavioral Basis for Smoking-Attributable Disease: A Report of the Surgeon Genera.

[29] Wisborg K, Henriksen TB, Secher NJ (2001). Maternal smoking and gestational age in twin pregnancies. Acta Obstet Gynecol Scand.

[30] Woods SE, Raju U (2001). Maternal smoking and the risk of congenital birth defects: a cohort study. J Am Board Fam Pract.

[31] Grillo E, Freitas PF (2011). Smoking and other pre-gestational risk factors for spontaneous preterm birth. Revista Brasileira de Saúde Materno Infantil.

[32] Kolas T, Nakling J, Salvesen KA (2000). Smoking during pregnancy increases the risk of preterm births among parous women. Acta Obstet Gynecol Scand.

[33] Kyrklund-Blomberg NB, Granath F, Cnattingius S (2005). Maternal smoking and causes of very preterm birth. Acta Obstet Gynecol Scand.

[34] Tuormaa TE (1995). The adverse effects of tobacco smoking on reproduction and health: a review from the literature. Nutr Health.

[35] Currie J, Moretti E (2007). Biology as destiny? short- and long-run determinants of intergenerational transmission of birth weight. J Labor Econ.

[36] Yerushalmy J, James G, Rosenthal T (1962). Smoking Habits of Father and Weight of Infant. Tobacco and Health.

[37] Martinez FD, Wright AL, Taussig LM (1994). The effect of paternal smoking on the birthweight of newborns whose mothers did not smoke. Group health medical associates. Am J Public Health.

[38] Rubin DH, Krasilnikoff PA, Leventhal JM, Weile B, Berget A (1986). Effect of passive smoking on birth-weight. Lancet.

[39] Lazzaroni F, Bonassi S, Manniello E, Morcaldi L, Repetto E, Ruocco A, Calvi A, Cotellessa G (1990). Effect of passive smoking during pregnancy on selected perinatal parameters. Int J Epidemiol.

[40] Mathai M, Vijayasri R, Babu S, Jeyaseelan L (1992). Passive maternal smoking and birthweight in a south Indian population. Br J Obstet Gynaecol.

[41] Office on S, Health (2001). Publications and Reports of the Surgeon General. Women and Smoking: A Report of the Surgeon General.

[42] Kramer MS (1987). Determinants of low birth weight: methodological assessment and meta-analysis. Bull World Health Organ.

[43] Moore ML, Zaccaro DJ (2000). Cigarette smoking, low birth weight, and preterm births in low-income African American women. J Perinatol.

[44] Shah NR, Bracken MB (2000). A systematic review and meta-analysis of prospective studies on the association between maternal cigarette smoking and preterm delivery. Am J Obstet Gynecol.

[45] Semba RD, Kalm LM, de Pee S, Ricks MO, Sari M, Bloem MW (2007). Paternal smoking is associated with increased risk of child malnutrition among poor urban families in Indonesia. Public Health Nutr.

[46] Kyaing NN: *Regional Situation Analysis of Women and Tobacco in South-East Asia*. New Delhi: World Health Organization, Regional Office for South-East Asia; 2004.

[47] Hulme PA, Blegen MA (1999). Residential status and birth outcomes: is the rural/urban distinction adequate?. Public Health Nurs.

[48] Bushy A (1998). Health issues of women in rural environments: an overview. J Am Med Womens Assoc.

[49] Hughes D, Simpson L (1995). The role of social change in preventing low birth weight. Future Child.

[50] Hillemeier MM, Weisman CS, Chase GA, Dyer AM (2007). Individual and community predictors of preterm birth and low birthweight along the rural-urban continuum in central Pennsylvania. J Rural Health.

[51] Luo ZC, Wilkins R, Kramer MS (2006). Effect of neighbourhood income and maternal education on birth outcomes: a population-based study. CMAJ.

[52] Parker JD, Schoendorf KC, Kiely JL (1994). Associations between measures of socioeconomic status and low birth weight, small for gestational age, and premature delivery in the United States. Ann Epidemiol.

[53] Mozurkewich EL, Luke B, Avni M, Wolf FM (2000). Working conditions and adverse pregnancy outcome: a meta-analysis. Obstet Gynecol.

[54] Baranski B (1993). Effects of the workplace on fertility and related reproductive outcomes. Environ Health Perspect.

[55] Ha E, Cho SI, Chen D, Chen C, Ryan L, Smith TJ, Xu X, Christiani DC (2002). Parental exposure to organic solvents and reduced birth weight. Arch Environ Health.

[56] Khattak S, G KM, McMartin K, Barrera M, Kennedy D, Koren G (1999). Pregnancy outcome following gestational exposure to organic solvents: a prospective controlled study. JAMA.

[57] Peck J, Alexander K, Gamm LD, Hutchison LL, Dabney BJ (2003). Maternal, Infant, and Child Health in Rural Areas. Rural Healthy People 2010: A Companion Document to Healthy People 2010.

[58] Bove CF, Olson CM (2006). Obesity in low-income rural women: qualitative insights about physical activity and eating patterns. Women Health.

[59] Gilpin EA, Pierce JP, Cavin SW, Berry CC, Evans NJ, Johnson M, Bal DG (1994). Estimates of population smoking prevalence: self-vs proxy reports of smoking status. Am J Public Health.

[60] Adegboye AR, Heitmann B (2008). Accuracy and correlates of maternal recall of birthweight and gestational age. BJOG.

[61] Catov JM, Newman AB, Kelsey SF, Roberts JM, Sutton-Tyrrell KC, Garcia M, Ayonayon HN, Tylavsky F, Ness RB (2006). Accuracy and reliability of maternal recall of infant birth weight among older women. Ann Epidemiol.

[62] Little RE (1986). Birthweight and gestational age: mothers’ estimates compared with state and hospital records. Am J Public Health.

